# Effect of coating with combined chitosan and gallic acid on shelf-life stability of Jeju black cattle beef

**DOI:** 10.5713/ab.23.0180

**Published:** 2023-10-31

**Authors:** Van-Ba Hoa, Dong-Heon Song, Kuk-Hwan Seol, Yun-Seok Kim, Hyun-Wook Kim, In-Seon Bae, Soo-Hyun Cho

**Affiliations:** 1 Animal Products Utilization Division, National Institute of Animal Science, RDA, Wanju 55365, Korea

**Keywords:** Chitosan, Coating, Discoloration, Gallic Acid, Lipid Oxidation, Shelf-Life

## Abstract

**Objective:**

Beef of Jeju black cattle (JBC) is considered as a healthy meat type due to its significantly higher unsaturated fatty acids (UFA). Lipid (e.g., UFA) is highly susceptible to oxidizing agents, which results in the quality deterioration and economic value loss of meat products. Therefore, development and application of novel preservative techniques is necessary to improve the shelf-life stability of high-UFA beef. The objective of this study was to assess the applicability of chitosan-based coatings in preservation of JBC beef.

**Methods:**

Different coating solutions: 2% chitosan alone, and 2% chitosan containing 0.1% or 0.3% gallic acid were prepared to investigate their applicability in preservation of fresh beef during storage. Jeju black cattle beef (2-cm thick steaks) were non-coated (control) or coated with the above coating solutions, placed on trays, over-wrapped with plastic film and stored at 4°C. The microbiological indices, color, total volatile basic nitrogen (TVBN) and lipid oxidation of the beef were investigated after 1, 10, and 21 days of storage.

**Results:**

Coating with 2% chitosan alone reduced the spoilage bacteria count, TVBN and thiobarbituric acid reactive substances levels in the beef compared with control during storage (p<0.05). Noticeably, coating with 2% chitosan containing 0.1% or 0.3% gallic acid was more effective on retardation of spoilage bacteria growth, lipid oxidation and discoloration in the beef compared to the chitosan coating alone over the storage period (21 days) (p<0.05).

**Conclusion:**

Taken together, the combined chitosan and gallic acid coating could be used as a bio-preservative technique in the meat industry.

## INTRODUCTION

Fresh meat products are highly perishable due to their low oxidative stability and susceptibility to microbial spoilage that causes discoloration, off-flavors and deteriorative texture [1]. Therefore, preservation plays a crucial role in controlling the spoilage and extending the shelf-life of the meat and meat products during distributing and marketing. Until now, numerous conventional techniques such as; pasteurization, refrigerating, freezing alone or in combination with packaging have been used to enhance the meat preservation [2]. The packaging is also a crucial tool to retain the quality and prolong the shelf-life of meat. For many decades, the synthetic plastics have become the most dominant packaging materials in the food industry due to the low-cost and convenience [3]. However, the synthetic plastics are non-degradable materials that are harmful to the environment [4]. Recently, the development and application of edible coatings/films have become the emerging packaging alternatives in the preservation of meat [5]. Edible coating materials are applied on meat surfaces that act as a barrier against microbial growth and physicochemical deteriorations [6]. The edible coatings have become popular in the meat industry because of their environment-friendly characteristics, low cost and ability to provide protection after the packaging has been opened [7,8]. Among the edible coating materials, chitosan (originated from crustacean shells) approved as a food additive in many countries, has ability to form film and bio-degrade, and exerts its excellent antimicrobial activity [5]. Therefore, chitosan has recently been used to preserve meat and meat products [8,9]. Researchers have also reported that the shelf-life of meat can be further extended by combining multiple preservation technologies; for instance; a combination of coating with packaging method [10,11]. Additionally, the incorporation of bioactive compounds (e.g., polylysine, fatty acid, essential oils) into coating materials also improves the shelf-life of meat products during storage [8,10,12].

Gallic acid is a water-soluble phenolic compound that is commonly found in fruits and vegetables. Researchers have proven that gallic acid possesses potent antioxidant and antimicrobial activities [13]. Gallic acid has been used as a preservative agent to extend the shelf-life of fruits [14]. Furthermore, incorporation of gallic acid into coatings/films further enhances its bioactivity and mechanical strength and oxygen barrier properties [15]. However, little attention has been paid to application of combined chitosan/gallic acid edible coating in the preservation of meat products.

Jeju black cattle (JBC) are commonly raised in Jeju Special Self-Governing Province of Korea. Compared to the other beef breeds (e.g., Hanwoo), the JBC is generally maintained at a smaller population size [16]. Historical documents reported that the JBC meat was considered as a great delicacy presented to the King in the Chosun era in some special occasions (e.g., New Year or Memorial day). Nowadays, Korean consumers consider the JBC beef as a healthy meat type due to its significantly higher unsaturated fatty acids (UFA) content (about 65%) compared to Hanwoo beef (about 58%) [17]. It is well known that lipid (e.g., UFA) is highly susceptible to degradation, and its oxidation usually results in quality deterioration (e.g., discoloration) and economic value loss of meat and meat products [18]. Due to its high UFA content as above mentioned, the JBC beef may be associated with a higher risk of lipid oxidation as well as quality deterioration during storage.

On the other hand, overwrapping is the most commonly traditional packaging at retails due to its convenience and giving the fresh meat with a desirable red color due to the existence of oxymyoglobin on its surface [19]. This method entails placing pre-cut pieces of meat on trays and manually pre-overwrapped with plastic film. However, overexposure of the meat to oxygen may promote spoilage bacterial growth and discoloration. In this context, a suitable preservation technique should be developed and applied to reduce these unexpected occurrences and increase the shelf-life of the high-UFAs meat types such as JBC beef. Thus, the aim of this study was to investigate the applicability of chitosan-based coatings in preservation of beef under aerobic packaging condition.

## MATERIALS AND METHODS

### Preparation of coating solution and its application

**Figure 1:**
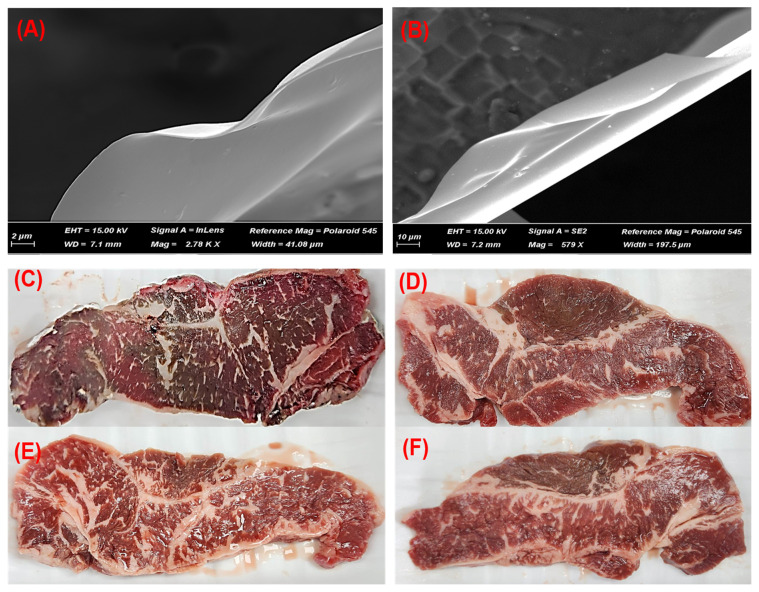
The images of scanning electron microscopy of dry-form of 2% chitosan (A) and 2% chitosan/0.3% gallic acid coating films (B); beef steaks non-coated (C), or coated with 2% chitosan alone (D), 0.1% gallic acid in 2% chitosan (E), and 0.3% gallic acid in 2% chitosan (F), were wrapped in plastic film and stored at 4°C for 10 days.

In the present study, different coating treatments were undertaken to evaluate the preservative effect of chitosan alone or combined chitosan/gallic acid coating on the shelf-life stability of JBC beef during refrigerated storage. For this purpose, chitosan (Sigma-Aldrich, St. Louis, MO, USA) at 2% (w/v) [8] and gallic acid (Sigma-Aldrich, USA) at 0.1% and 0.3% (w/v) were used. The concentrations of gallic acid set in the present study were the referred to level used by Fang et al [11], who reported that incorporation of 0.2% or 0.4% gallic acid into chitosan coating exhibited a strong inhibitory effect against lipid oxidation and spoilage bacteria growth in fresh pork under modified atmosphere packaging condition during storage. The chitosan coating solution was made by adding 2% chitosan into 1% (v/v) acetic acid solution (Sigma-Aldrich, USA), and then stirred for 24 h at room temperature. Thereafter, 0.1% and 0.3% gallic acid was incorporated into the chitosan solutions which were then homogenized for 5 min. The final pH of coating solutions was adjusted to 5.8 with sodium bicarbonate. The morphological properties of coating solutions were also analyzed. Prior to the analysis, about 0.5 mL of each coating solution was applied on silicone, dried at 4°C for 24 h, coated with platinum and then analyzed using a scanning electron microscopy Supra 40 VP instrument (Zeiss Co., Oberkochen, Germany). We observed that the coating solutions used in the present study were capable of film forming (Figure 1).

*Longissimus lumborum* (LL) muscles (at 48 h post-mortem) collected from the left carcass side of 32 months-old JBC steers (n = 10) were used. The muscles were cut into 2-cm thick steaks after removing visual fat tissues from the exterior. For coating treatment (T), the steaks were immerged in the 2% chitosan solution alone (T1), or 2% chitosan with 0.1% gallic acid (T2), and 2% chitosan with 0.3% gallic acid (T3) for 30 s to completely cover them. The coated samples were placed in a cooling room (4°C) for 30 min for drying. Non-coated samples were used as a control. Thereafter, the non-coated and coated steaks were placed on white foam trays (1 steak/tray) and overwrapped with 0.15 μm polyvinyl chloride film. The sample trays (n = 40; 10 per treatment) were stored in a cooling room at 4°C, and the shelf-life stability including: microbiological quality, total volatile basic nitrogen (TVBN), thiobarbituric acid reactive substances (TBARS) and color were investigated on day 1, 10, and 21.

### Shelf-life measurements

#### Microbiological indices

On the completion of each storage period, a 10-g of each sample was weighed immediately after removing the plastic film, placed in sterile bag containing 90 mL saline solution and homogenized for 1 min using a Stomacher. Following a tenfold progressive dilution, approximately 1.0 mL of each sample was spread on Aerobic Count Agar or Lactic Acid Bacteria Count Agar plates (3M Health Care; St. Paul, MN, USA) for the total aerobic plate count (APC) and lactic acid bacteria (LAB) enumeration. For enumeration of *Pseudomonas* spp. approximately 0.1 mL of each sample was spread on pseudomonas agar base (Oxoid Ltd., Hants, England) supplemented with pseudomonas selective agar supplement (RS0103). All the plates were incubated at 37°C in an incubator for 48 h. The results were expressed in logarithms of number of colony forming units (log_10_ cfu/g).

#### Meat color measurement

After the microbiological sampling was completed, the color was measured using a Minolta Chroma Meter CR-400 with a D65 illuminant*C and 2° observer (Minolta Camera, Osaka, Japan). The Commission Internationale de l’Eclairage (CIE)L* (lightness), a* (redness), and b* (yellowness) values were measured directly on the sample surface without removing the coating. Five measurements were taken for each sample. Additionally, the discoloration in the non- and coated meat samples was also calculated using the measured a* values, and expressed as a percentage of a* value loss after each storage period.

#### Total volatile basic nitrogen

The TVBN content formed in the meat samples during storage was measured in accordance with procedure of Seong et al [20] with suitable modifications. Briefly, duplicate aliquots (5.0 g) of each sample were taken and homogenized with 45 mL distilled water at 11,000 ×rpm for 30 s. Next, the samples were filtered with Whatman filter paper (No.1), and the filtrates were used for the TVBN measurement. For this, 1.0 mL of 0.01 N boric acid and 100 μL of Conway reagent (0.066% methyl red: 0.066% bromocresol green, 1:1) were added to the inner space while, 1.0 mL sample was added to the outer space of Conway tool. After adding 1.0 mL of 50% (w/v) K_2_CO_3_ solution into the outer space, the Conway tool was sealed immediately and kept at 37°C for 2 h. Finally, the boric acid was titrated with various volumes of 0.02 N H_2_SO_4_ solution until it turned violet. The TVBN content was calculated using the following formula:


$$\text{TVBN }(\text{mg}/100\;\text{g})=\frac{\left[(\text{a}-\text{b})\times \text{F}\times 28.014\right]}{\text{S}}\times 100$$

Where, a is the volume (mL) of added H_2_SO_4_ into the sample, b in is the volume (mL) of added H_2_SO_4_ into the blank, S is the weight (g) of sample, F is factor of the used H_2_SO_4_.

#### Thiobarbituric acid reactive substances

The TBARS assay was conducted using the method as described in our previous study [12] to determine the lipid oxidation in the meat samples during storage. For this assay, duplicate aliquots (5.0 g) of each sample were blended in a homogenizer (Ultra-Turrax T25B) at 11,000×rpm for 15 s in 15 mL distilled water, 50 μL saturated butylated hydroxyanisole and 30 mL of thiobarbituric acid (0.02 M)/trichloroacetic acid (15% w/v) (TBA/TCA at 1:1 ratio). After heating at 90°C in a water bath for 15 min, the samples were cooled on ice for 20 min, and then centrifuged at 3,000×g for 10 min. The supernatants were carefully taken and their absorbance values were attained using a spectrophotometer (Infinite M200; Tecan Ltd., Mannedorf, Switzerland) at 531 nm. Results were reported as mg malondialdehyde (MDA) equivalent/kg sample (MDA/kg).

### Statistical analysis

The Statistical Analysis System (SAS) package (SAS Institute, Cary, NC, USA, 2018) was used to analyze the data. A two-way analysis of variance of SAS was used to analyze the data. In the statistic model, coating and storage time were considered as main effects while, the obtained data was considered as the variables. The data were analyzed by using the general linear model of SAS. Means were compared using Duncan’s multiple range test. Differences among mean values were set at p<0.05. Data were expressed as mean±standard deviation.

## RESULTS AND DISCUSSION

### Effect on the microbiological indices

Fresh meat products are easily contaminated with microorganisms from various sources such as; animals (e.g., skin and intestine etc.) and slaughterhouse environment (e.g., equipment, handlers and knives) [1]. The growth of spoilage bacteria is among the main processes involved the quality loss and shelf-life shortening of meat [21]. The effect of coating on the microbial counts in JBC beef during storage is presented in Table 1. After 1 day of storage, the APC, LAB, and *Pseudomonas* spp. counts ranged from 2.82 to 2.94 log_10_ cfu/g, 1.62 to 1.72 log_10_ cfu/g and 1.94 to 1.98 log_10_ cfu/g, respectively, and no differences in the counts occurred between the non-coated and coated meat samples (p>0.05). This result indicated the similar sanitation status as well as a low initial load of microorganisms in all the beef samples. After 10 days of storage, the APC, LAB, and *Pseudomonas* spp. counts showed a significant (p<0.05) difference between the control and coating treatment. Particularly, the control (non-coating) displayed the highest APC (4.60 log_10_ cfu/g), LAB (4.38 log_10_ cfu/g), and *Pseudomonas* spp. (4.62 log_10_ cfu/g) counts, followed by the T1 (APC, 3.40 log_10_ cfu/g; LAB, 2.11 log_10_ cfu/g; and *Pseudomonas* spp., 3.51 log_10_ cfu/g), T2 and T3 (APC, 2.96 log_10_ cfu/g; LAB, 1.83 log_10_ cfu/g; and *Pseudomonas* spp., 2.88 log_10_ cfu/g) (p<0.05). After 21 days of storage, the same trend of bacteria growth was observed in the control and coating treatments as those at the 10-day storage. Over 21 days of storage, the total APC increased by 4.73, 1.29, 0.67, and 0.65 log_10_ cfu/g, in the control, T1, T2, and T3, respectively. The statistical analysis showed that the APC progressively increased in the control, T1 and T2 over the storage periods, but it did not increase in the T3 during the first 10 days and only increased thereafter. Similarly, the count of *Pseudomonas* spp. only increased during the first 10 days and then decreased thereafter. Thus, over 21 days of storage, the *Pseudomonas* spp. increased by 4.46 and 0.48 log_10_ cfu/g in the control and T1, respectively. Interestingly, the count of *Pseudomonas* spp. in the chitosan/gallic acid coated-samples (T2 and T3) at 21-day was similar or lower than the initial count (day 1). This results signified that the combined chitosan/gallic acid coating showed a stronger inhibitory effect against the growth of bacteria in the beef samples during storage.

It is recognized that under the aerobic packaging condition, APC and *Pseudomonas* spp. are the major bacterial spoilers in meat products during refrigerated storage [22]. Especially, *Pseudomonas* spp. metabolizes glucose, free amino acids and proteins resulting in spoilage of the meat products as soon as their counts reach 6 log_10_ cfu/g [23]. Additionally, it is recommended that the maximum limit of APC in fresh meat types should not exceed 5 to 7 log_10_ cfu/g [24]. Based on our results it may be said that under aerobic packaging condition, the non-coated JBC beef was spoiled after 21 days of refrigerated storage. Whilst, the beef samples coated with chitosan alone or combined chitosan/gallic acid displayed the APC that was much lower than the recommended limit for the fresh meat as above mentioned.

Similar to the current findings, Duran and Kahve [8] and Cheng et al [9] reported that coating with 2% chitosan effectively inhibits the bacteria growth in vacuum-packaged beef during refrigerated storage. Compared with APC reported in beef at 1 day of storage by these authors, our result showed a lower level. This could be related to the differences in hygienic status (initial bacteria loads) of the meat samples used between the studies. The main mechanism behind the antimicrobial activity of chitosan has been attributed to the interaction between the positive charge of free amino groups on the chitosan with the negative charge on cell membranes, resulting in leakage of intracellular contents and cell death [25]. Furthermore, gallic acid is a phenolic acid containing 3 hydroxyl groups which cause the changes in permeability, rupture and pore formation in cells and death of microorganisms [13]. In the present study, the higher inhibitory effect against the spoilage bacteria growth in the chitosan/gallic acid-coated beef samples compared with the chitosan coating alone during storage could be attributed to the synergistic antimicrobial activities by both the chitosan and gallic acid. The antimicrobial activity of incorporated gallic acid in the beef samples also showed a dose dependent manner. Our results suggest that the incorporation of 0.1% gallic acid into chitosan coating could be sufficient to inhibit the microbial growth under the current experimental conditions. Supporting our results, Fang et al [11] reported that adding 0.2% or 0.4% gallic acid into chitosan increases the inhibitory effect against the growth of bacteria in modified atmosphere packaged pork during storage.

### Effect on lipid oxidation

In the presence of oxygen (e.g., aerobic packaging condition), UFAs are the main substrate determining the extent of lipid oxidation which causes the loss of nutritional value, discoloration and quality deterioration in meat and meat products [18]. The TBARS value is widely used as an indicator for judging the extent of lipid oxidation in meat and meat products. The effect of coatings on the TBARS value in the beef during storage is presented in Table 2. The initial TBARS value (day 1) of beef samples ranged from 0.35 to 0.39 mg MDA/kg, without differences between the non- and coated meat samples (p>0.05). However, after 10 and 21 days of storage, the control (non-coating) displayed the highest TBARS value followed by T1 (chitosan coating alone) whilst, the beef coated with chitosan containing 0.1% (T2) or 0.3% (T3) gallic acid displayed the lowest TBARS values (p<0.05). We observed that the TBARS value in the control sharply increased up to 2.58 mg MDA/kg (increased by 2.19 mg/kg) after 21 days of storage. The TBARS level increased in the non-coated samples in the present study was higher than level (increased by 1.66 mg/kg) reported in Hanwoo LL muscle under the same packaging condition after 21 days of storage by Hoa et al [12]. This indicates that the rate of lipid oxidation was faster in the JBC beef compared with Hanwoo beef. The meat samples coated with chitosan alone (T1) showed a slower increasing rate of TBARS value (only increased by 1.15 mg MDA/kg after 21 days of storage) with increased storage time compared with the control (p<0.05). The ability of chitosan to form film on meat surface, leading to reduced exposure of the meat to oxygen, and its metal ions chelating, have been proposed as the main mechanisms underlying the antioxidant activity of this material [26]. Interestingly, the incorporation of gallic acid (T2 and T3) showed a stronger effect on retarding the lipid oxidation in the beef during storage compared with those coated with chitosan alone (p<0.05). As shown in Table 2, after 21 days of storage the TBARS level only increased by 0.22 and 0.17 mg/kg in the T2 and T3, respectively. We also observed that raising the concentration of gallic acid to 0.3% had a stronger effect on delaying the lipid oxidation for instance, the TBARS value only increased in the T3 during the first 10 days and did not increase thereafter. This is probably because of gallic acid that is able to stop the oxidation reaction [27].

### Effect on total volatile basic nitrogen content

The TVBN value has widely been used as freshness indices for meat products. It is well known that volatile nitrogenous compounds are generated in meat products as a result of the degradative processes of non-proteins and proteins by endogenous proteolytic enzymes and spoilage bacteria [22]. The TVBN content in the beef samples during storage as affected by the coatings is shown in Table 3. After 1 day of storage, significant difference in the TVBN content occurred between the control and coating treatment; the control had a higher level (11.24 mg/100 g) than the T1 or T2 and T3 (8.99 to 9.93 mg/100 mg) (p<0.05). A similar trend was seen after 10 and 21 days of storage; the control presented a higher value (22.10 and 26.22 mg/kg, respectively) compared with T1 (15.55 and 18.54 mg/100 g, respectively) or T3 (12.74 and 15.92 mg/100g, respectively) (p<0.05). Thus, the TVBN value of the non-coated samples at 10-day storage was higher than the maximum standards (15 to 20 mg/100 g) recommended for the fresh meat products in some countries such as China or Korea [22]. As expected, the addition of gallic acid enhanced the inhibitory effect against the TVBN production in the beef samples after 10 and 21 days of storage compared with the chitosan coating alone (p<0.05). In general, the TVBN content in all the samples significantly increased with increased storage time, but the fastest rate was seen in the control, followed by the T1, T2, and T3. However, no differences in the TVBN content occurred between the two gallic acid concentrations (0.1% and 0.3%) over the storage time. This phenomenon was also observed in the lipid oxidation (Table 2) as discussed above, suggesting that incorporation of 0.1% gallic acid into the chitosan is sufficient to inhibit the lipid oxidation and TVBN production in the beef samples. The results indicating a lower TVBN value in the coated beef samples could be attributed to the antimicrobial effects of chitosan [25] and gallic acid [13] because the spoilage bacteria significantly contribute to the production of VBN via the degradation of non-proteins and proteins in meat [22]. Supporting the present results, Cheng et al [9] reported that coating with chitosan effectively reduces the TVBN content in beef during storage. Fang et al [11] also showed that addition of 0.2% gallic acid to chitosan further enhanced the inhibitory effect against TVBN production in pork.

### Effect on color stability

Color is the most important primary criterion by which the consumers judge the quality (freshness and wholesomeness) and acceptability of meat [28]. The mean values of color traits in the control and coated samples during storage are presented in Table 4. The initial (day 1) values of L* (lightness), a* (redness), and b* (yellowness) showed no difference between the control and coating treatments (p>0.05). These values were almost similar to those reported for the LL muscles from Hanwoo and Chikso steers [29]. After 10 days of storage, the L* values also showed no differences between the non-and coated samples (p>0.05), however, the a* values were significantly (p<0.05) lower in the non-coated samples compared with the coated samples. After 21 days of storage, the samples coated with chitosan containing gallic acid (T2 and T3) exhibited the highest L* and a* values whereas, the control showed the lowest values (p<0.05). On the other hand, the storage time affected the color traits of all the samples. As presented in Table 5, the a* values were decreased by 57.43% in the control after 21 days of storage (p<0.05). This indicates a severe discoloration of the non-coated meat samples (Figure 1). The chitosan-coated samples (T1) also showed a decrease in the a* values (approximately by 47.41%, Table 5) after 21 days of storage (p<0.05). This means that coating with chitosan alone was insufficient to protect the beef from discoloration under the current experimental condition. However, when the gallic acid was incorporated the protective effect of chitosan coating against the discoloration in the beef samples during storage was significantly enhanced. Particularly, after 21 days of storage, the a* values were decreased only by 38.06% and 25.11% in the chitosan/0.1% gallic acid (T2) and chitosan/0.3% gallic acid (T3), respectively. A bright red color is an important trait reflecting the freshness of meat, and it is mainly contributed by oxymyoglobin pigment [28]. The decrease in a* values is attributed to the oxidation of oxymyoglobin to metmyoglobin. In the present study, the more severe discoloration in the non-coated meat samples might be resulted from a faster rate of oxymyoglobin oxidation whereas, coating with chitosan alone or combined chitosan/gallic acid could reduce the permeation rate of oxygen into the muscle tissue, leading to the more color stability due to the retarded oxymyoglobin oxidation. Thus, the addition of gallic acid into chitosan more effectively protected the beef from discoloration under aerobic packaging condition. Similar to the current finding, Alirezalu et al [10] and Hoa et al [12] reported that the addition of active compounds (e.g., short chain fatty acids or ɛ-polylysine and rosemary essential oil) to chitosan further enhance the protective effects on the discoloration in beef during storage.

## CONCLUSION

This study, for the first time assessed the effects of combined chitosan/gallic acid coating on shelf-life stability of beef under aerobic packaging condition. The coating with chitosan containing 0.1% or 0.3% gallic acid successfully inhibited the growth of spoilage bacteria, and delayed the lipid and proteins oxidations, and discoloration in the JBC beef during refrigerated storage. Based on the results obtained from this study, it is suggested that the combined chitosan and gallic acid coating could be developed and used as bio-preservative technique to improve the shelf-life of aerobically-packaged meat products. Further study is required to evaluate whether the coating treatment affects the eating quality properties (e.g., taste and flavor) of beef.

## Figures and Tables

**Table 1:** Microbiological quality of coated Jeju black beef under aerobic packaging condition during storage (1, 10, and 21 d)

Treatment^1)^	Aerobic plate count (log_10_ cfu/g)			Lactic acid bacteria (log_10_ cfu/g)			*Pseudomonas* spp. (log_10_ cfu/g)		
		
	1 d	10 d	21 d	1 d	10 d	21 d	1 d	10 d	21 d
Control	2.94±0.07^C^	4.60±0.66^aB^	7.67±1.81^aA^	1.67±0.12^C^	4.38±1.10^aB^	4.71±1.17^aA^	1.96±0.08^C^	4.62±0.70^aB^	6.42±2.00^aA^
T1	2.84±0.03^C^	3.40±0.05^bB^	4.13±0.04^bA^	1.62±0.06^C^	2.11±0.04^bB^	2.32±0.02^bA^	1.98±0.10^C^	3.51±0.03^bB^	2.46±0.05^bA^
T2	2.86±0.06^C^	3.19±0.05^cB^	3.53±0.04^cA^	1.67±0.15^B^	1.94±0.03^cA^	2.10±0.04^cA^	1.95±0.03^B^	3.10±0.02^cA^	1.97±0.01^cB^
T3	2.82±0.03^B^	2.96±0.02^d^^B^	3.47±0.02^cA^	1.72±0.19^B^	1.83±0.03^cAB^	1.97±0.07^d^^A^	1.94±0.10^B^	2.88±0.07^d^^A^	1.69±0.06^d^^C^

1) Control, non-coating; T1, coating with 2% chitosan alone; T2, coating with 0.1% gallic acid in 2% chitosan solution; T3, coating with 0.3% gallic acid in 2% chitosan solution.

a–c Means in a same column with different superscripts differ significantly (p<0.05).

A–C Means in a same row with different superscripts differ significantly (p<0.05).

**Table 2:** Thiobarbituric acid reactive substances level (mg malondialdehyde/kg) in the coated Jeju black beef under aerobic packaging condition during storage

Treatment^1)^	Storage time		

	1 d	10 d	21 d
Control	0.39±0.03^C^	1.59±0.32^aB^	2.58±0.17^aA^
T1	0.38±0.03^C^	0.97±0.02^bB^	1.53±0.16^bA^
T2	0.35±0.01^C^	0.49±0.03^cB^	0.57±0.06^cA^
T3	0.37±0.04^B^	0.48±0.02^cA^	0.54±0.02^cA^

1) Control, non-coating; T1, coating with 2% chitosan alone; T2, coating with 0.1% gallic acid in 2% chitosan solution; T3, coating with 0.3% gallic acid in 2% chitosan solution.

a–c Means in a same column with different superscripts differ significantly (p<0.05).

A–C Means in a same row with different superscripts differ significantly (p<0.05).

**Table 3:** Concentration (mg/100 g) of total volatile basic nitrogen (TVBN) in the coated Jeju black beef under aerobic packaging condition during storage

Treatment^1)^	Storage time		

	1 d	10 d	21 d
Control	11.24±1.23^aC^	22.10±0.92^aB^	26.22±1.36^aA^
T1	8.98±0.71^bC^	15.55±1.11^bB^	18.54±1.85^bA^
T2	8.99±1.01^bC^	13.67±1.11^cB^	16.67±1.80^cA^
T3	9.93±0.85^bC^	12.74±1.16^cB^	15.92±0.46^cA^

1) Control, non-coating; T1, coating with 2% chitosan only; T2, coating with 0.1% gallic acid in 2% chitosan solution; T3, coating with 0.3% gallic acid in 2% chitosan solution.

a–c Means in a same column with different superscripts differ significantly (p<0.05).

A–C Means in a same row with different superscripts differ significantly (p<0.05).

**Table 4:** Color traits of coated Jeju black beef under aerobic packaging condition during storage (1,10, and 21 d)

Treatment^1)^	L*			a*			b*		
		
	1 d	10 d	21 d	1 d	10 d	21 d	1 d	10 d	21 d
Control	35.23±2.35^A^	35.76±2.83^AB^	33.31±1.51^bB^	22.01±1.53^A^	13.98±2.28^bB^	9.37±1.28^cC^	11.17±0.68^A^	8.03±1.41^bcB^	7.38±1.26^B^
T1	36.52±4.46^A^	33.73±1.53^AB^	33.38±3.43^abB^	21.98±3.04^A^	15.72±1.21^abB^	11.56±3.79^bcC^	11.09±1.39	7.58±0.87^c^	7.21±0.77
T2	36.47±2.31	35.63±2.19	35.59±4.97^ab^	22.26±1.40^A^	16.96±2.48^aB^	13.75±2.97^abC^	11.27±0.93^A^	8.87±1.38^abB^	7.36±0.56^B^
T3	36.36±2.49	35.84±2.99	35.12±2.29^a^	20.55±2.89^A^	17.54±1.60^aB^	15.39±2.98^aB^	11.01±1.63^A^	9.77±0.89^aA^	7.66±1.26^B^

1) Control, non-coating; T1, coating with 2% chitosan only; T2, coating with 0.1% gallic acid in 2% chitosan solution; T3, coating with 0.3% gallic acid in 2% chitosan solution.

a–c Means in a same column with different superscripts differ significantly (p<0.05).

A–C Means in a same row with different superscripts differ significantly (p<0.05).

**Table 5:** Loss percentage (%) of a* (redness) values of coated Jeju black beef under aerobic packaging condition during storage (1, 10, and 21 d)

Treatment^1)^	Storage time		

	1 d	10 d	21 d
Control	0	36.48±2.16^aB^	57.43±3.28^aA^
T1	0	28.48±1.12^abB^	47.41±2.19^abA^
T2	0	23.60±1.17^abB^	38.06±1.76^bc^^A^
T3	0	14.65±1.32^bB^	25.11±2.11^c^^A^

1) Control, non-coating; T1, coating with 2% chitosan only; T2, coating with 0.1% gallic acid in 2% chitosan solution; T3: coating with 0.3% gallic acid in 2% chitosan solution.

a,b Means in a same column with different superscripts differ significantly (p<0.05).

A,B Means in a same row with different superscripts differ significantly (p<0.05).
